# Childhood autism in a 13 year old boy with oculocutaneous albinism: a case report

**DOI:** 10.1186/1752-1947-2-56

**Published:** 2008-02-22

**Authors:** Muideen O Bakare, Nkeiruka N Ikegwuonu

**Affiliations:** 1 Child and Adolescent Unit, Federal Neuro-Psychiatric Hospital, New Haven, Enugu, Enugu State, Nigeria

## Abstract

**Introduction:**

Hypomelanotic skin disorders like tuberous sclerosis and hypomelanosis of Ito that present with multiple systemic manifestations have been reported in association with childhood autism. Oculocutaneous albinism is another hypomelanotic skin disorder that rarely presents with multiple systemic manifestations. It is infrequently reported in association with childhood autism when compared to tuberous sclerosis and hypomelanosis of Ito.

**Case presentation:**

This article reports a case of co-morbid childhood autism and oculocutaneous albinism in a 13-year old boy from Nigeria in Sub-Saharan Africa.

**Conclusion:**

The observation in this case report and in two previous reports which documented association between oculocutaneous albinism and childhood autism both in the affected individuals and families of individuals with childhood autism, raises the question of a possible genetic and clinical association between oculocutaneous albinism and childhood autism. More family and genetic studies into the relationship between oculocutaneous albinism and childhood autism is desirable. This may provide useful clues into the etiology, prevention and management of childhood autism as well as oculocutaneous albinism.

## Introduction

Several inherited medical and psychological disorders have been reported in association with childhood autism and many of these disorders are providing valuable information on the role played by genetics in the etiology of childhood autism. Hypomelanotic skin disorders like tuberous sclerosis and hypomelanosis of Ito that present with multiple systemic manifestations have been reported in association with childhood autism.

Oculocutaneous albinism is another hypomelanotic skin disorder that rarely presents with multiple systemic manifestations. It is infrequently reported in association with childhood autism when compared to tuberous sclerosis and hypomelanosis of Ito.

## Case presentation

The patient is a 13-year-old Igbo boy from the South-Eastern region of Nigeria in Sub-Saharan Africa. He had been a resident of a destitute home managed by a Catholic Reverend Sister in Enugu, Nigeria. The destitute home provides habitation for mainly abandoned children and vagrant psychotic patients. The first psychiatric service contact with the patient had been during a community mental health service sponsored by the Rotary Club of Independence Layout, Enugu, Nigeria in which the authors volunteered. The patient was abandoned in a refuse dump a few days after delivery and he had grown up with other abandoned children who found habitation in the destitute home because his parents could not be traced.

### Medical and psychiatric history

The patient was born with oculocutaneous albinism and on growing up he had been a child in a world of his own. He rarely played with other children in the destitute home and failed to develop like other children of his age. He was unable to develop speech and incapable of verbal communication; he only screamed sharply if in distress or in need of attention. He avoided eye to eye contact and he often appeared to be looking into space focusing on an unseen object. He failed to reciprocate any social gestures extended to him by his multiple care-givers over the years. He did not turn around if his name was called and he was most of the time preoccupied with playing with his fingers. He often snatched away other children's meal and snacks after finishing his own. Presently at the age of 13 years the patient could not utter a word, only shouting and screaming sometimes without apparent reason. He did not respond to instructions and appeared distant when attempts were made to interact with him. He however often responded to the word 'take', especially if the individual interacting with him was holding a biscuit or any other snacks, which he usually snatched away forcefully and ate voraciously. Associated behavioral problems included destructive tendencies, screaming without apparent reason and running around the destitute home in a circle, which gave him delight as he almost always resisted any attempt to stop him and often required forceful intervention to redirect him.

There was no history of any major medical illness that could influence normal neurological development in the patient during childhood and throughout the period of his stay at the destitute home.

### Developmental and social history

Gross motor development was said to have been normal when compared to other children in the destitute home. Developmental impairments were restricted to the areas of communication, cognition and social interaction. The patient had not been exposed to any form of schooling and he had had no social interaction outside the destitute home where he had lived for thirteen years.

### Family history

No information is available about family history.

### Mental state evaluation

He appeared to be oblivious of his environment and was staring into space, focusing on an unseen object. He did not respond to any questions or instructions during interaction and made no speech of his own. His attention and concentration were impaired during interaction. His attention was however drawn with the word 'take' to which he snatched away forcefully the biscuit from the examiner's hand and ate voraciously as if the biscuit would be taken away from him if he was not fast enough. After this, he was once again in his own world. There was no abnormal motor activity.

### Physical examination and psychological investigation

**Figure 1:**
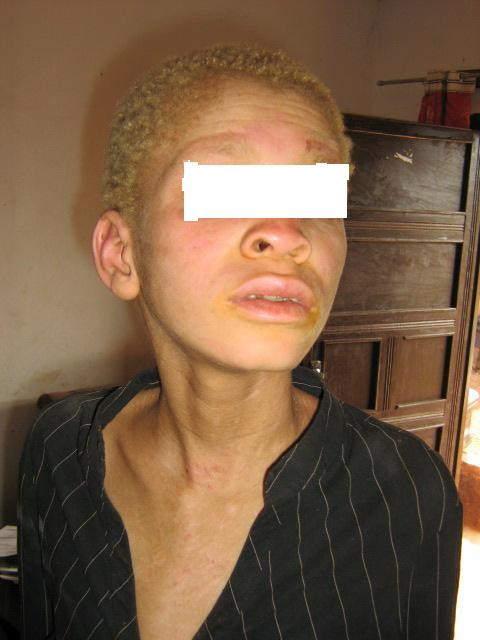
Showing patient's skin color.

**Figure 2:**
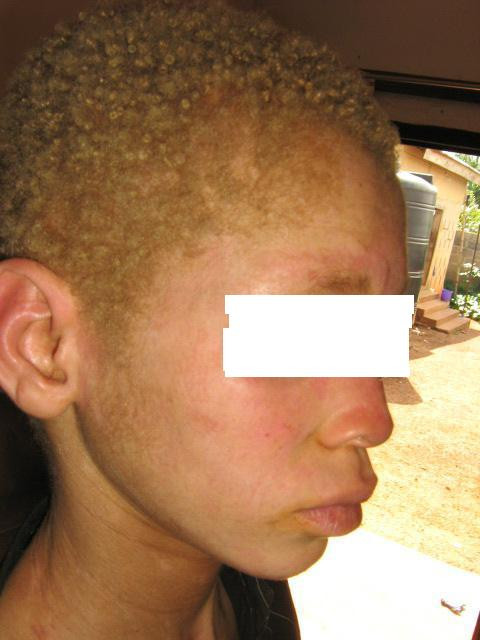
Showing patient's skin and hair color.

Physical examination revealed a young boy with features of oculocutaneous albinism (Figures 1 and 2). There were no other specific skin lesions. He was small in stature for his age. Gross examination of the Central Nervous System (CNS) revealed no hearing or vision impairment. There was no motor abnormality or sensory deficit. Examinations of other systems were essentially normal.

Formal Intelligent Quotient (I.Q) test was not carried out on this patient because of the confounding variation that socio-cultural influences have on standardized I.Q tests. However, empirical judgment based on interaction with the patient in the milieu of his socio-cultural environment showed that he was severely retarded with mental age estimated at around 3 to 4 years.

### Diagnosis and treatment

Based on the history, physical examination and clinical interaction with the patient, co-morbid diagnoses of oculocutaneous albinism (E 70.3) and childhood autism (F 84.0) with severe mental retardation (F 72) were made based on World Health Organization (W.H.O) International Classification of Diseases, 10^th ^Edition (ICD – 10) [1]. Identified problems in the patient were communication impairment, poor social interaction, and behavioral problems characterized by unwarranted screaming, unruly behavior of snatching away other children's snacks, hyperactivity that was often displayed by running around the destitute home in a circle and destructive tendencies. He was referred to the Child and Adolescent Psychiatry unit of Federal Neuro-Psychiatric Hospital, New Haven, Enugu, Nigeria for further evaluation and management.

A multi-disciplinary approach to the management of children with autism is desirable, however there are limited facilities for children with autism in a third world country like Nigeria.

However, the behavioral problems were managed with oral Haloperidol 2.5 mg daily on which the patient had been on for three weeks with reduction in hyperactivity and unruly behavior toward other children.

## Discussion

Childhood autism, also known as autistic disorder or infantile autism is a neuro-developmental condition that is characterized by impairment in social interaction, impairment in communication and restricted or stereotyped patterns of behavior and interest usually manifested before the age of 3 years [2]. The median prevalence world wide is between 0.04 and 0.05 percent [2]. The condition was first described in 1943 by Leo Kanner [3].

Several inherited medical and psychological disorders have been reported in association with childhood autism and many of these disorders are providing valuable information on the role played by genetics in the etiology of childhood autism [4-6].

### Inherited hypomelanotic skin disorders and childhood autism

Some inherited hypomelanotic skin disorders that present with multiple systemic manifestations have been reported in association with childhood autism. These include tuberous sclerosis [6-8] and hypomelanosis of Ito [4,9]. That these two hypomelanotic skin disorders associated with childhood autism show neuro-cutaneous and multi-systemic manifestations would point to possible dysfunctional migration of ectodermal and mesodermal cell precursors during embryogenesis in the etiology of these conditions and possibly leading to the neuro-developmental problems that characterize childhood autism. The pattern of chromosomal aberrations found in hypomelanosis of Ito and the polymorphic nature of the condition have led to the belief that hypomelanosis of Ito syndrome is a descriptive term rather than a true syndrome [10]. If this belief is anything to go by, then the genetic basis and etiological process of the dysfunctional migration in ectodermal and mesodermal cell precursors in this condition during embryogenesis leading to possible neuro-developmental problems observed in childhood autism could be heterogeneous.

Further studies on dysfunctional maturation and differentiation of ectodermal and mesodermal cell precursors during embryogenesis in hypomelanotic skin disorders in general may be needed to unravel the pathophysiology of childhood autism.

### Oculocutaneous albinism and childhood autism

Oculocutaneous albinism is a hypomelanotic skin disorder. It is inherited in an autosomal recessive process and it rarely presents with multiple systemic manifestations such as have been found in other inherited hypomelanotic skin disorders like tuberous sclerosis and hypomelanosis of Ito associated with childhood autism. Oculocutaneous albinism is infrequently reported in association with childhood autism when compared to tuberous sclerosis and hypomelanosis of Ito.

However, Rogawski et al [11] had reported co-morbidity of oculocutaneous albinism and childhood autism in two boys and Delong [12] in a recent description of families of individuals with childhood autism had noted additional feature of oculocutaneous albinism in some families in addition to major affective disorder and special talents. Going by the observation of this present case report and the report of these two previous reports in the literature [11,12], the question arises whether childhood autism has any genetic and clinical relationship with oculocutaneous albinism.

## Conclusion

Further studies on dysfunctional maturation and differentiation of ectodermal and mesodermal cell precursors during embryogenesis in inherited hypomelanotic skin disorders that have been associated with childhood autism are needed. More family and genetic studies into the exact relationship that could exist between oculocutaneous albinism and childhood autism are also desirable. These may provide useful clues into the etiology, prevention and management of childhood autism as well as oculocutaneous albinism.

## Competing interests

The author(s) declare that they have no competing interests.

## Authors' contributions

Both authors participated in the management of the patient. The first author prepared and revised the manuscript. Both authors approved the final draft of the manuscript.

## Consent

We thanked Reverend Sister Mary-Anthony Chukwubuikem who is in charge of the Maryland destitute home in Enugu, Nigeria for giving her written informed consent for publication of this case report and the accompanying images. A copy of the written consent is available for review by the Editor-in-Chief of this journal.
